# Genealogy of the Meningococcus and Gonococcus

**Published:** 1917-04

**Authors:** 


**Genealogy of the Meningococcus and Gonococcus.** Miss. Valley Med. Jour., Feb., Chas. E. Barnett of Ft. Wayne. The author describes a case of gonorrhoea of severe type, in a 19 year old farmer's boy, infected along with 13 others from the same source. Supra-pubic cystotomy was performed on account of the large amount of pus, high temperature and septic reaction. Staphyloccus albus was isolated from catheterizedurine, later colon bacillus a gram negative coccus considered Neisser's. Tuberculin and Wassermann reactions were negative, micrococcus catarrhalis, geniococcus and meningococcus strongly positive. Gonoccus and diplococcus from patient's urine, strongly positive. Except as stated, antigens and cultures were obtained from a distance. The author raises the question whether the meningococcus, as suggested by the positive reaction, is not genealogically related to the gonococcus.

